# Comment on “ERK and p38MAPK combine to improve survival in patients with BRAF mutant colorectal cancer”

**DOI:** 10.1038/s41416-018-0279-3

**Published:** 2018-10-09

**Authors:** Burak Uzunparmak, Ibrahim H. Sahin

**Affiliations:** 1 The University of Texas MD Anderson Cancer Center, Houston, TX USA; 2 Emory University School of Medicine, Atlanta, GA USA

**Keywords:** Tumour immunology, Prognostic markers

We read the interesting paper by Roseweir et al.,^1^ which explored the biomarker utility of ERK and p38MAPK in colorectal cancer patients carrying a BRAF mutation. The authors identified phosphorylation of ERK and p38MAPK as a surrogate measure to predict better outcome in BRAF-mutant mismatch repair (MMR)-proficient colorectal cancer patients, who were diagnosed with stage III disease. The prognostic value for combined nuclear pERK and p38 score remained significant after multivariate analysis. The authors did not observe this association in MMR-deficient (MMR-D) BRAF-mutant colorectal cancer patients.

BRAF mutations in colorectal cancer are strongly associated with CpG Island methylator phenotype (CIMP), which leads to gene silencing due to methylation of promoter areas of certain genes.^2^ CIMP phenotype is known to be associated with sporadic microsatellite instability-high (MSI-H) colon cancers due to frequent methylation of MLH-1.^2,3^ Approximately 60% of loss of MLH-1 expression loss is attributable to BRAF mutations.^3^ To date, several other studies suggested that more than half of the BRAF-mutant colorectal cancer patients carry the MSI-H phenotype due to loss of MLH-1 function,^4–6^ particularly in patients with early stage disease. However, in the paper by Roseweir et al., the frequency of MSI-H tumours in BRAF-mutant stage I−III colorectal cancer patients was only 16% (27/165, Figure 2), suggesting this cohort may not represent the general BRAF-mutant colorectal cancer population (as a side note, although the authors reported 165 BRAF-mutant patients, the Kaplan Meier analyses in Figure 2 included a total of 166 patients). Relatively lower incidence of this important subset of patients might have also impacted the multivariate analysis conducted by the authors. Lastly, in the subgroup analysis of colorectal cancer patients, strong nuclear pERK1/2 pattern was significantly (*P* = 0.01, Table 3) less frequent in patients with MMR-D tumours. The combined nuclear pERK/p38 score was also relatively less detected in MMR-D cases with a borderline statistical significance (*P* = 0.065, Table 2) suggesting there may be an inverse association between MMR-D status and the pERK/p38 score. Given MMR-D colorectal cancers patients constitute a relatively larger segment of BRAF-mutant patients which is associated with better outcomes due to MSI-H phenotype,^4^ reported inverse relation between MSI-H and pERK/p38 score may generate further challenges regarding the applicability of this score.

Overall, although the authors uncovered a potential important biomarker in BRAF-mutant colorectal cancer patients with MSS tumours, the title of the article does not appear to reflect the authors’ findings, as the association they found was limited only to MMR-proficient BRAF-mutant patients. This constitutes a relatively smaller subset of BRAF-mutant colorectal cancer patients, particularly considering that the association was significant only in stage III tumours. Moreover, these findings may need to be further confirmed in another cohort as the relationship between BRAF mutation and MMR-D reported in this article appears to be weak and is not consistent with other reported literature as discussed above.
